# On the Chemical Relations of Urinary Calculi

**Published:** 1840-04

**Authors:** 


### ART. III.

*De Chemicis Calculorum Vesicariorum Rationibus.* Scripsit E. A. SCHARLING, A.A., L.L., M. Chemiæ Lector.—*Hauniæ*, 1839. 4to, pp. 52.

*On the Chemical Relations of Urinary Calculi.* By E. A. SCHARLING, Lecturer on Chemistry, &c.—*Copenhagen*, 1839.

IT has happened to us, on several occasions, during the last few years, to find ourselves challenged by the merit of Inaugural Dissertations, both British and Foreign, to rank them among our *Books*, and to notice them as such. The work of M. Scharling belongs to this class; and claims our attention both for the importance of its subject and the ability with which it is treated.

In the account which the author gives of the labours of those who have preceded him, it is a matter of congratulation to an Englishman to find, that his own countrymen have taken by far the largest share in the chemical investigation of calculi. Some suspicion of the nature of urinary concretions appears to have been entertained as early as the fifteenth century, but it was Scheele who, in 1776, first examined these bodies in a scientific manner; although, according to him, they all contained the same principles. Bergmann improved upon the experiments of Scheele, and first announced the presence of oxalic acid in calculi, although, according to the author, we are to suppose that he produced it artificially, by acting on lithic acid with nitric acid. Scopoli and Bernoulli, finding also what they considered to be the acid of sugar (oxalic acid) in calculi, recommended that the use of saccharine, and other substances capable of producing that acid, should be avoided.

In 1786, Tychsen, professor of chemistry in Copenhagen, discovered in a calculus, given to him by Winslöw, phosphoric acid combined with lime; and in 1793, Fourcroy, after having confirmed some of the experiments of Scheele and Bergmann, announced the occasional presence of the phosphates of ammonia and soda (p. 3). In 1797, Wollaston proposed a classification of these bodies, founded on a series of his own experiments; and this, with slight modifications, has continued in use until the present time. The history of calculi may be considered to have been rendered very complete by the researches of Marcet, who added to Wollaston's varieties the xanthic oxide and the fibrinous calculi. It is unnecessary here to enumerate the various additions to the subject made by Brande, Henry, and Prout, in England, and Liebig and Wöhler in Germany, for these must be sufficiently known to the profession.

The author, after remarking that so long as the chemical characters of calculi remained unknown, it was hopeless to look for any good result from medical treatment, proceeds to speak of the physical and chemical properties of these concretions. In giving a brief outline of his observations, we shall only select those points which appear to us new, and a knowledge of which is likely to be of practical utility.

1. *Form.* Calculi are generally round or oblong; those which are of large size are often pyriform. Angular calculi are commonly small, and angularities are generally observed where *several* of these concretions are contained in the bladder. Sometimes they have a tetrahedral or cubic form, specimens of which are figured in the author's plates.

2. *Size and specific gravity.* The size of calculi is subject to great variation. Thus they may weigh from a few grains to several pounds. Out of 126 specimens in the Royal Surgical Museum of Copenhagen, examined by the author, more than one half weighed between half an ounce and two ounces. In estimating the weight of calculi, attention is properly directed to a source of fallacy not commonly noticed by writers on this subject. Thus, if a calculus be weighed soon after removal, its weight will be much greater than after the lapse of a few months. This is what we might expect, from the large quantity of water which these bodies contain, and which slowly passes off by evaporation. This loss of weight even goes on, in some instances, for several years. A singular observation of Professor Jacobson's is referred to, as an illustration: A calculus examined on the 9th of July, 1832, weighed two ounces and twenty grains; on the 11th July, it had lost twenty grains; and, going on slowly decreasing, it had lost on the 12th August, 1833, *fifty-six grains.* The weights of calculi cannot, therefore, be compared, except in those cases where the time at which the weighing is performed is accurately recorded.

Fourcroy found the sp. gr. of calculi to vary from 1·213 to 1·976. The author considers this statement to be generally correct, but in one instance a calculus examined by him had a sp. gr. of 2·014. In estimating the sp. gr., it is necessary that the calculus should be deprived of the air which, from its porosity, it always contains, after it has been exposed. Thus the calculus must be placed for some hours under water, or in vacuo, or it may be boiled in water; the last plan is objectionable, since a portion may become thereby dissolved.

3. *Surface.* Our attention to this is so far important, that the actual constituents of the crust of calculus may be sometimes determined by an inspection of the surface. The angular prominences on the so-called mulberry calculus indicate oxalate of lime, but this calculus is sometimes smooth. The compound phosphate of ammonia and magnesia often forms large foliated crystals, and the subphosphate of lime has commonly a smooth chalky surface. The lithic acid variety has either a smooth surface, or, if it be rough and uneven, the prominences are obtuse and rounded, not acute, like those on the mulberry calculus. (p. 9.) Where more than one calculus is contained in the bladder, these superficial characters are modified or lost by friction. Deep fissures are often seen on calculi, sometimes filled up by septa of membranous matter, or by urinary deposits of a different nature. The chemical composition of an entire calculus can rarely be deduced from an examination of its surface, since the body may be made up of very different materials.

4. *Colour.* This is a point to be attended to in judging of the nature of calculi. Calculi are generally met with of three colours: a dull yellow or *brown,* indicative of lithic acid or the lithates; a *dull purple,* the oxalate of lime; and the third and most frequent *white,* which indicates the phosphates and carbonates. The colours are never so well seen as in the recent calculi; the fifty coloured specimens delineated in the plates have been taken under these circumstances.* Calculi may present different colours on section. This will, of course, be the case where there is a difference of composition.

5. *Odour.* Calculi often retain an unpleasant odour, if they have not been washed after removal. This is due to the presence of decomposing animal matter locked up in the interstices, such as urine, mucus, &c. When rubbed or rasped, they give out the empyreumatic odour of bone, under the same circumstances. Fourcroy remarked that the mulberry calculus had the odour of spermaceti. This may have been accidental.

The structure of calculi is commonly lamellar, the different layers sometimes easily separating from each other; but the ammoniaco-magnesian phosphate variety seldom admits of this, owing to its softness and brittleness. To show the lamellæ, Berzelius recommends that the calculus should be polished by grinding it with water and the powder of calculi; but the author prefers the simple use of a brush with water. The lamellæ are generally arranged with considerable regularity around a central part, which acts as a nucleus. They are often of different composition, and appear to have been performed at various times and under different states of the system.

The nucleus is the most important part of the calculus, since this seems to be often the primary cause of the production of the calculus. (" Quæ sæpe prima causa calculi formati, æstimari potest.") Although the nucleus may have the same chemical composition as the other parts, it is generally denser. That the nucleus derives its origin from the kidneys the author thinks probable, from the fact that small renal calculi are frequently passed by those subject to calculous disease, before any symptoms of stone in the bladder appear; and also because such individuals often suffer from the most severe pains, when the calculi descends from the kidneys into the bladder. Sometimes substances mechanically introduced into the bladder from without are known to act as nuclei for the calculous deposit. Instances of this kind are to be found recorded by most surgical writers.

Occasionally two nuclei are found; two specimens of this variety are figured, and in some very rare cases more than two. An admirable drawing is given (fig. 38), in which there are four nuclei; this calculus was composed entirely of phosphate of lime. Sometimes there is no nucleus, but there is a space in the centre, filled by membrane, or lined by crystals; at other times the calculus is perfectly hollow, of which a specimen, in the possession of Professor Jacobson, is figured (fig. 5). This, probably like most others presenting this singular conformation, was not closed, but had fissures leading down to the centre from various parts of the surface. The nucleus on which the calculous matter had become deposited was perhaps originally soft, and had become, in the progress of time, removed through these fissures.

* We must here do the author the justice to say, that the drawings are the most correct, in regard to colour and form, which we have ever seen. Here and there, we meet with the objection which applies to coloured lithographs generally, namely, that the blackness of the ink tends to drown the tints.

Having thus given the author's account of the *physical* characters of calculi, we shall next proceed to examine his view of the *chemical* relations of these bodies. He enumerates twenty-two different substances which have, up to the present time, been discovered by chemists as constituting the varieties of urinary calculi. It is needless to mention these: many exist already formed in the urine, others are purely extraneous, as clay and the phosphate of iron. He himself has even found mica in one calculus; "but substances of this kind are to be regarded as of accidental occurrence, and not constituents of calculi. Many of the substances detected by chemists are not very soluble in water; it is easy to conceive that these may be readily deposited; but it is difficult to understand how very soluble salts should be separated in a crystalline form, unless by supposing that, like urea, which is occasionally found in small quantity in calculi, they proceed from that portion of urine which is closely locked up in the calculus at the time of extraction." (p. 13.)

The author's classification of calculi does not differ from that in common use:

1. *Lithic acid and the lithates.* More than three parts of all calculi are formed of this acid, either free or united to bases. In the free state, the acid is never pure, being mixed with more or less albumen or mucus and colouring matter. The chemical properties of these calculi are very accurately detailed: the action of nitric acid is properly relied on as the most satisfactory test. Nitric acid forms a colourless solution with lithic acid, leaving on evaporation a rich red residue, the colour of which immediately disappears on the addition of water, according to Berzelius; but the author did not find that this effect took place until after the lapse of several hours. His friend Jacobson adopts the following neat method in the application of the test: He evaporates cautiously in a watch-glass the nitric acid solution of lithic acid, until it has become inspissated; he then inverts this watch-glass over another, containing a few drops of ammonia, when the peculiar pinkish red colour, indicative of lithic acid, immediately appears. This is, no doubt, a red colouring matter generated simultaneously with purpuric acid. Instead of adopting this simple view of the changes, a sect of modern innovators have sought to complicate the subject, by assuming the production of such substances as alloxan, allaxantin, purabonic acid, and ammonia. This mystical trifling in science seems to have made no impression on our author, who, like a sober investigator, prefers plain facts and well-known names to crude hypotheses and a perpetual change of nomenclature.

Lithic acid may play a very important part in the animal economy: thus it has been lately discovered, that from it may be formed allantoin or allantoic acid—a principle detected by Vauquelin in the allantoic fluid of the cow.

The alkaline lithates are successively examined as constituents of calculi, and the means of identifying them are well described. The lithate of ammonia is known from those of potash and soda by the fact that it is entirely decomposed and dissipated by heat, while the two latter substances leave a residue of their respective carbonates. The author has met with two compounds of lithic acid and ammonia; that containing the larger proportion of acid abounding in the excrement of many varieties of birds. Lithic acid forms a neutral and an acid compound with lime, the latter being separated as a bilithate, on adding muriatic acid to the neutral combination.

According to the author's experience, calculi consisting chiefly of lithate of ammonia are rare. There was only one specimen in the whole collection referred to; this was taken from a young girl, and the ammoniacal lithate, when it occurs, is chiefly found in young subjects. The analysis of this calculus is very simple; but we do not see the necessity of pursuing the plan recommended by the author, namely, to separate the lithic acid by muriatic acid, before testing for ammonia. The mere heating of the powdered calculus with a small quantity of solution of potash is sufficient.

The lithate of lime sometimes accompanies the phosphate or the oxalate of that base. We must refer to the treatise for the means adopted to separate the lithate from these salts; the processes are simple and satisfactory. The lithate of magnesia is rare, but three specimens are referred to, two of which have already been described, as having the singular forms of a cube and a tetrahedron. As the analysis of this calculus is not given in works on chemistry, we here subjoin the author's description, premising that he has found this lithate more soluble in water than that of lime.

"A portion of the calculus is boiled in water, the solution filtered, and muriatic acid added, on cooling, to the filtered liquid. This separates the lithic acid, which may be obtained on a filter; and now, by boiling the clear liquid with potash, a white precipitate falls down, which is proved to be magnesia, by the facility with which it dissolves in sulphuric acid, as also by its dissolving, although more slowly, in muriate of ammonia." (p. 18.)

2. *Xanthic oxide.* We look in vain, in most modern works on calculi, for a satisfactory account of this variety, which, nevertheless, was discovered a long time since by Dr. Marcet. Some writers have even been disposed to doubt the existence of such a calculus, as a distinct species. Wöhler and Liebig have lately confirmed the researches of Marcet and Stromeyer, on its chemical constitution. This body is composed of the same elements, in the same proportions, as lithic acid, with the exception of its containing one equivalent less of oxygen. A better name has, therefore, been proposed for it, viz., *lithic oxide*, instead of that it now bears, and which is derived from its colour. The following are its principal characters:

"Its surface is smooth, and of a brownish colour, but in some parts earthy and white. It has a reddish brown fracture. It is lamellated in a concentric form, the lamellæ being easily separable from each other, without presenting any fibrous or crystalline union. There is no nucleus distinguishable from the rest of the mass. Its hardness is about equal to that of some of the more compact specimens of lithic acid calculi. It acquires by friction a waxy lustre. It is dissolved by warm nitric acid, without the evolution of any gas; and on evaporation, it leaves a lemon-coloured residue. It never produces with nitric acid the pink-coloured matter which is generated under the same circumstances with lithic acid." (p. 19.)

The last statement is opposed to the results of Marcet; but the author thinks that in the calculi examined by that excellent chemist there may have been some traces of lithic acid present. Again:

"To render the examination more complete, a portion was dissolved in caustic alkali; the solution thus obtained had a greenish yellow colour, resembling that of bile. It was somewhat opaque, and very difficult to filter. Carbonic acid was passed into this solution, until the alkali was converted to bicarbonate, by which means the xanthic oxide was entirely precipitated under the form of a white powder. After washing, the precipitate did not retain the smallest trace of alkali, in which respect it differs from lithic acid; for this body, thus treated, is never precipitated in a pure state, but always as an alkaline lithate, while another portion of acid is retained in the alkaline solution.

"The xanthic oxide is more soluble in caustic ammonia than lithic acid. On evaporation, it leaves a yellowish foliaceous residue, retaining traces of ammonia. The nature of the yellow mass obtained on evaporating the nitric acid solution of this substance is not known. Xanthic oxide is dissolved by strong sulphuric acid, and water does not precipitate it from this acid solution, in which respect it again differs from lithic acid. It is not soluble in muriatic and oxalic acid, in which, among other properties, it differs from *cystine.* When distilled in the dry way, it resembles lithic acid, in yielding a large quantity of hydrocyanic acid. An empyreumatic matter at the same time results, having a peculiar odour, resembling that of burnt horn. When sublimed, carbonate of ammonia but no urea is generated." (p. 20.)

Such is the description given by the author, by which, we think, professional men will in future be easily able to identify this species of calculus. Its rarity may, however, be inferred from the fact that there was not a single example of it among the museum specimens which he examined.

3. *Ammoniaco-magnesian phosphate.* This calculus was first discovered by Wollaston in the human bladder. Its white crystalline structure and spongy character at once indicate it. Potash expels the ammonia, precipitates the magnesia, and forms an alkaline phosphate. By calcination, ammonia is volatilized, and phosphate of magnesia remains. The test recommended for the fixed base is calcining it when moistened with the nitrate of cobalt, which brings out a rose-red colour; but there are other and better ways of proving the presence of magnesia. This calculus is easily soluble in dilute sulphuric acid, by which it is known from the phosphate of lime; the muriatic and acetic acids dissolve it. The muriatic solution, evaporated to dryness and still further heated, yields muriate of ammonia.

4. *Subphosphate of lime mixed with the compound phosphate of ammonia and magnesia.* This is the most frequent variety of calculus, after lithic acid. The author, in admitting that we are indebted to Marcet, Tennant, and Wollaston, for a knowledge of its chemical properties, says that its composition was known to his countryman, Tychsen, in 1786, eleven years before Wollaston's essay appeared. This calculus is commonly called the fusible variety, since, from its very compound nature, it easily fuses when heated. Its characters will be found detailed in most chemical works. The compound phosphate is sometimes mixed with the carbonate of lime; acetic acid affords a ready means of separating the constituents of this calculus, the subphosphate of lime remaining undissolved. (p. 22.)

5. *Phosphate of lime.* This is known from the subphosphate by the fact that, when heated to a high temperature, it fuses into a pearly mass. The author gives a quantitative analysis of one of these calculi, to show that it was neutral in composition; but we are not satisfied that his method would effectually separate all the lime. He reserves the process for determining the presence of phosphoric acid, until he speaks of the means of separating the mixed phosphates in a subsequent part of the essay. As this process is omitted in many works treating of the analysis of calculi, we here subjoin it, recommending dilute nitric acid to be used as the solvent, in preference to the concentrated acid employed by the author: "Dissolve a portion of the phosphate in dilute nitric acid; add a few drops of nitrate of silver, and then cautiously neutralize the excess of nitric acid by a weak solution of ammonia; phosphoric acid is known to be present by the immediate production of the yellow subphosphate of silver." (p. 35.)

6. *Oxalate of lime.* This salt forms the hardest calculi; their peculiar form serves to distinguish them: they are generally mixed with organic and other substances. In one, quantitatively analyzed by the author, organic matter and water formed thirty per cent. of its weight, and it contained besides about six per cent. of phosphate of lime. The author relates the method of detecting the base, but he omits to mention how the experimentalist may discover the acid. There are several processes; but one, which we have not seen noticed in books, is, to dissolve a portion of the calculus in dilute nitric acid; add nitrate of silver, and then gradually neutralize with ammonia: the white oxalate of silver is thrown down, known by its unchangeableness on boiling, and by its detonation at a moderate heat. If the oxalate be mixed with phosphate, the usual plan may be followed, of boiling the calculus with an alkaline carbonate.

The oxalate of lime calculus is sometimes met with in a perfectly crystalline state. Marcet saw this in three cases, and in another instance Wollaston observed that the crystals had an octohedral form.

7. *Carbonate of lime.* This is rarely found as a calculus in the human subject, although not unfrequently in animals. Its analysis is very simple, but the author adverts to a possible fallacy, which may mislead the experimentalist. In adding a dilute acid to any calculus, bubbles of air are often observed to escape; this must not be mistaken for carbonic acid; it is nothing more than the evolution of air which adheres to the surface of all porous solids. The presence of a sulphuret will lead to effervescence on the addition of an acid, but this is easily known by a salt of lead. The author has observed the escape of sulphuretted hydrogen from the urinary concretions of the ox during analysis. Carbonate of lime forms the principal mass of calculi found in animals.

Traces of carbonate of magnesia have been occasionally found in urinary calculi.

The description of cystic oxide or cystine is borrowed from Wollaston, through Berzelius. The occasional presence of silica, oxide of iron, and oxalate, and benzoate of ammonia in calculi, is next noticed; but no light is thrown upon the manner in which the first two of these substances become mixed with the more common constituents.

In a pathological point of view, it is a matter of great importance to determine which of the varieties of calculi are of the most frequent occurrence. Tables of this kind have been drawn up, and are attached to the collections of most museums; but it is probable, as M. Scharling remarks, that many of these are erroneous, from the circumstance of the outer crust only having been chemically examined. (p. 30.) Sometimes it is wished that the form of the stone should not be impaired by section, in which case it is obviously absurd to place it in any class, from the chemical composition of its crust. We cannot but admit the correctness of this view, when we reflect on what has been already said respecting the structure of calculi. We fully coincide with the author in the opinion that every calculus should be classified according to the chemical composition of its *principal mass.* In a table of 155 calculi examined by him, he has given the composition, in one column, of the nucleus, in a second, of the principal mass, and in a third, of the outer crust or cortex. If such a rule as this were followed by future observers, different collections might allow of instructive comparisons; whereas now it is often a matter of much uncertainty to compare them. The results of M. Scharling's observations are as follows: "In 94, the great bulk of the calculus was made up of lithic acid, or about sixty per cent. In 23, oxalate of lime, or fifteen per cent. In 25, the mass was composed of the phosphates, or about sixteen per cent." (p. 31.) It is worthy of remark, that in 73, the outer crust was formed of the phosphates; so that, had the examination been confined to this part only, the phosphatic calculi would have amounted to forty-seven per cent., instead of sixteen per cent. It is necessary to observe, that many of the lithic acid and oxalate of lime calculi are really invested by a crust of the phosphates: this is especially the case in the larger varieties of all calculi. The proportions contained in other museums, especially those of England, are then referred to; they do not in general materially differ from those obtained by the author.

The next part of the treatise contains matter deeply interesting to those who wish to engage in chemical researches on the properties of these bodies. It is customary, in most treatises, to give the chemical characters of the varieties of calculi individually, by which the student is led to suppose that the analysis is a very simple operation; but a great practical difficulty meets him in his first attempts, in the fact that the varieties are commonly blended together; hence the chemical reactions peculiar to one substance are obscured by the presence of another. When a calculus is cut through, portions of the different lamellæ should be separately examined; and when these do not exist, when we have only a confused mass for analysis, it will be necessary to pursue a certain series of experiments, if we wish to spare ourselves much labour, and obtain a satisfactory result. M. Scharling's is the only treatise which we have yet seen, wherein these rules are clearly and succinctly described. For an account of these, as well as for the best means of separating from each other all the usual mixtures of calculi, a subject so embarrassing to a beginner, we must refer to the work itself. The rules are evidently derived from long experience, and accurate observation.

There are some sound remarks on the deposition and increase of calculous matter in the bladder. Foreign substances accidentally introduced and left in the organ, are often observed to become incrusted with the phosphates. This has been explained by supposing that, in such cases, the urine abounded in those saline matters, and that there must have existed a constitutional tendency to calculous disease. The author, however, considers, and with great probability, that the change in the chemical constitution of the urine may really have been due to the irritation produced by the presence of a foreign body in the organ; an opinion borne out by the fact, that such individuals had never manifested any symptoms of calculus previously to the foreign substance becoming lodged in the bladder. The great difficulty is, to explain the cause of the formation of renal calculi; for these are often the nuclei of the deposits formed in the bladder. So again, the separation of a small quantity of fibrin or mucus may become a nucleus, around which the urinary salts are more or less rapidly deposited. The increase of the stone may depend greatly, in the author's view, on the state of health of the individual; and the thickness of the lamellæ may serve as a criterion of the vicissitudes of health and disease in a person so affected, the thickest lamellæ being deposited while the individual is in a bad state of health.

A very curious part of the enquiry relates to the singular alternations in the chemical characters of the deposits: the nucleus, the body, and the cortex being perhaps different from each other. It is often observed that this transition is not abrupt, but gradual, indicating slow and secret changes in the constitution, by diet, medicine, or variable state of health. Thus M. Scharling has remarked, in several mulberry calculi, the transition from lithic acid to oxalate of lime to take place through a deposit of the lithate of lime. (p. 41.) The effect of diet in calculous disorders deserves to be more deeply studied than it has hitherto been; we should probably hereby discover the source of many of these singular chemical changes. When in one part of a calculus the lamellated character abruptly ceases, it is probable, as Crosse imagines, that the calculus may have become adherent by this part to the parietes of the bladder. On the whole, the increase in the size of a calculus bears much analogy to the slow growth of crystals placed in saline solutions.

*On chemical solvents.* Since the composition of calculi has been known, various remedies of a chemical nature have been recommended. The alkalies and their carbonates have been employed to neutralize the lithic acid diathesis; but the author thinks they may be also efficaciously employed against the oxalate of lime and the phosphates. We agree with him, that an alkaline carbonate can decompose the oxalate of lime; but we should hardly have supposed, although he gives it as the result of his own observation, that the natural heat of the body would have sufficed for this decomposition. We have found that a heat of 212° was required for some minutes, to bring about a partial interchange of acids and bases; for under no circumstances is the decomposition complete. Thus, then, in the most favorable case, we substitute a carbonate of lime for an oxalate of lime calculus; but he thinks the carbonate is susceptible of removal, by the subsequent use of carbonated water. In his opinion, the alkaline carbonates will easily decompose the phosphates, especially the basic and neutral phosphates of lime. The alkali should be used in the state of bicarbonate. (p. 43.)

It is difficult to explain how it is that the carbonates of lime and magnesia have had a beneficial effect in calculous disorders, where those of potash and soda have failed. The author thinks, with Wöhler, that any vegetable neutral salt of potash or soda may be substituted for the carbonates, since in the animal body those salts are actually converted to carbonates. The mineral biborate of soda (borax), which is a more powerful solvent of lithic acid than the carbonate, has been used in calculous affections; but this does not become transformed into carbonate like the vegetable salts. The acetate of soda was tried, by M. Scharling, as a solvent for lithic acid, but he found it to have less power than the biborate; yet, when lithic acid was mixed with the basic phosphate of lime, the solvent power of that salt was greater than that of borax, so far as the calcareous phosphate was concerned. (p. 44.) Either of these salts should be exhibited in the form of injection. Acetate of potash resembles the acetate of soda, but the acetate of magnesia has no more power than the carbonate of that alkaline earth. Acids, both mineral and vegetable, have been used with variable effects; the latter have been preferred.

M. Scharling answers the objections which have been made to the use of chemical antidotes; and he thinks he has had good physical evidence of the solvent action of these remedies on calculi which he has examined. The surface has appeared cellular, as if worn away or dissolved. The figures of five calculi are referred to by him, in confirmation of this view, although perhaps other explanations might be brought forward to account for this cellular state of the surface. On the whole, there is much difference of opinion on the subject of chemical remedies; but we agree with the author, that those who advocate their use have far greater reason on their side than those who entirely reject them. He recommends the remedies to be employed by injection, taking care that the solutions are not so strong as to excite irritation of the bladder; but he very judiciously adds, these are only remedies for the *local* mischief: to remove the disease itself, the system must be put under active medical treatment.

We thus complete our review of M. Scharling's essay, which, although published in an unpretending form, contains more useful and practical information on the subject of which it treats than many voluminous treatises. We could have wished that it had appeared in any other language than Latin, since this is but ill adapted for the diffusion of scientific knowledge in modern times; yet the author has perhaps taken the best means he could adopt, his own tongue, the Danish, being but little understood by professional men. As a literary composition, it may, perhaps, be destitute of what is called classical polish, but it possesses qualities which have far greater value in our eyes, namely, clearness and simplicity.

